# Value of Magnetic Resonance Imaging for Diagnosis of Dentigerous Cyst

**DOI:** 10.1155/2016/2806235

**Published:** 2016-09-27

**Authors:** Antonione Santos Bezerra Pinto, André Luiz Ferreira Costa, Neiandro dos Santos Galvão, Thásia Luiz Dias Ferreira, Sérgio Lúcio Pereira de Castro Lopes

**Affiliations:** ^1^Department of Morphology, Faculty of Medicine, Federal University of Ceara, Fortaleza, CE, Brazil; ^2^Department of Orthodontics and Radiology, University of São Paulo City (UNICID), São Paulo, SP, Brazil; ^3^Piracicaba Dental School, State University of Campinas (UNICAMP), Piracicaba, SP, Brazil; ^4^Department of Diagnosis and Surgery, São José dos Campos Dental School, São Paulo State University (UNESP), São José dos Campos, SP, Brazil

## Abstract

Odontogenic cysts have a high prevalence in the dental clinic population, with dentigerous cyst being one of the most frequent ones and whose aetiology involves accumulation of fluid between the reduced enamel epithelium and the crown of an unerupted tooth. In the diagnostic process of these lesions, one should consider complementary imaging exams such as conventional radiography and computed tomography, which are commonly used for providing anatomical information on the tissues compromised by the lesion, but not on the nature of it. Magnetic resonance imaging (MRI) scans are noninvasive modalities which, due to their unique acquisition characteristics, can provide distinct information on the nature of the lesion. This study reports on a case of dentigerous cyst in the mandible of a 9-year-old patient, documented by means of different imaging modalities. MRI played an important role in both diagnosis of the lesion and differential diagnosis between neoplastic lesions presenting similar imagenological behaviour under other techniques of radiography.

## 1. Introduction

A considerable number of lesions can compromise the dentomaxillofacial complex. Among these, one can highlight tumours and cysts of odontogenic origin as they are highly prevalent in the dental clinic population. The majority of the gnathic cysts are lined by odontogenic epithelium—the reason by which they are termed odontogenic cysts [1, 2].

Dentigerous cyst is the most common odontogenic cyst originating from the separation of the pericoronal follicle from the unerupted tooth, with prevalence of about 20 percent among all cysts lined with epithelium in the maxillae [3].

In the diagnostic process of these lesions, one should consider imaging exams, such as the complementary modalities due to mainly their easiness of access, acquisition, and use of relevant information for conducting the diagnostic process [4]. Imaging techniques (panoramic, occlusal, and periapical radiographs) allow localisation of lesions, but they are not specific [5]. In the case of dentigerous cyst, it is necessary a three-dimensional view of the cortical bone and assessment of the cyst content to make a better diagnosis [6, 7].

In addition to the conventional radiographic techniques, the cone beam computed tomography (CBCT) is a remarkable modality as it provides accurate information regarding location, delimitation, and effects of lesions on the surrounding structures, since the resulting images are shown with no overlapping and distortion, which allows us to characterise the structures three-dimensionally. Nevertheless, despite the actual contributions, these exams have significant limitations compared to MRI, with exposure to X-ray radiation being the main one [8].

Recent research has shown that MRI provides information which are beyond the image quality, not exposing the patient to the harmful effect of ionising radiation and thus helping determining a precise diagnosis both spatially and anatomically for conducting the treatment. The determination of the content of the lesions is another advantage consistent with MRI compared to other imaging modalities [6, 9].

The aim of this study is to discuss on several diagnostic imaging modalities by means of a case report of a dentigerous cyst, emphasising the singular role of MRI in the analysis and treatment planning for such a lesion.

## 2. Case Presentation

**Figure 1:**
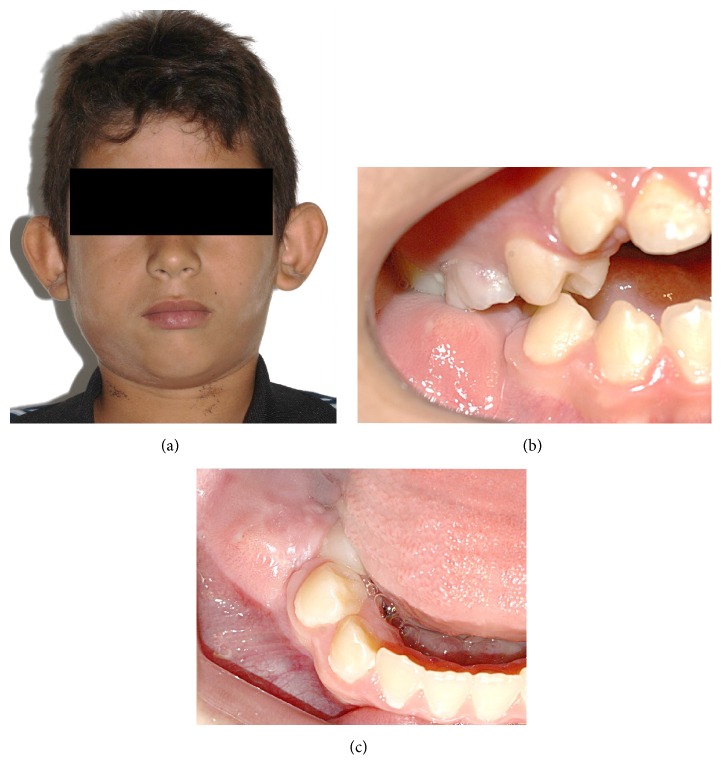
Clinical aspect of the lesion. Facial asymmetry (a) and tumefaction in the right posterior region of the mandible. Front (b) and upper (c) views.

A 9-year-old male patient was referred to our clinics for evaluation of the presence of asymptomatic tumefaction, hard on palpation, in the right posterior region of the mandible, resulting in a facial asymmetry. Intraoral examination showed evidence of lingual displacement of tooth #45 as well as presence of root remnants of tooth #46, including a solid mass lined by mucosa of normal appearance in the region corresponding to the entire alveolar crest extending occlusally (Figure 1).

**Figure 2:**
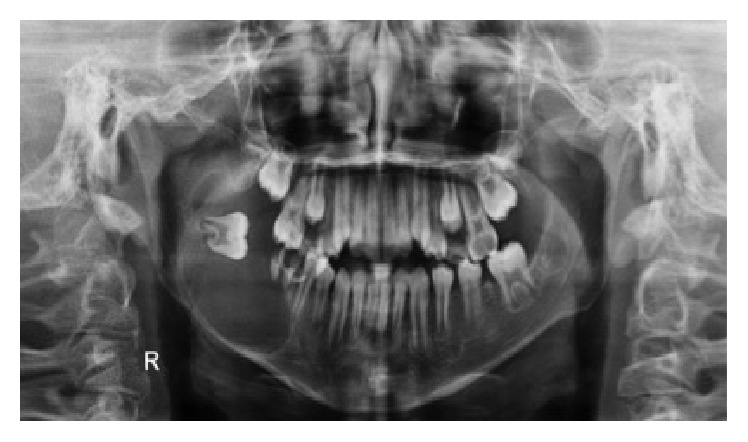
Panoramic radiograph: radiolucid image of right mandibular body and ramus, with involvement of the included tooth #47.

Panoramic radiograph was performed and showed a radiolucid, unilocular image of precisely corticalised limits involving tooth #47, which was in horizontal position and projecting towards the ipsilateral region of the mandibular ramus, with the crown medially positioned. This lesion extended from the apical region of tooth #45 to the corresponding mandibular ramus, with a very thin mandibular base encompassing the root remnants of tooth #46 (Figure 2). The radiographic appearance was suggestive of benign odontogenic lesion. Among the diagnostic hypotheses raised, dentigerous cyst, ameloblastoma, and keratocystic odontogenic tumour were considered.

**Figure 3:**
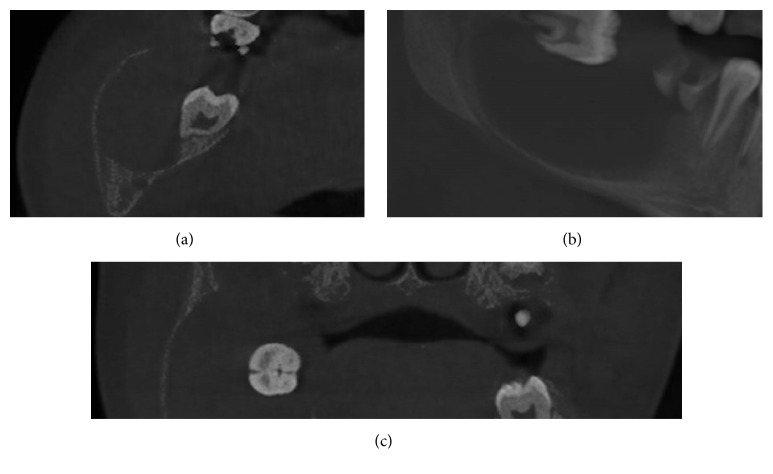
CBCT images showing axial (a), sagittal (b), and coronal (c) slices demonstrating the expansive aspect of the lesion and its internal homogeneous appearance.

Cone beam computed tomography (GXCB-500™, powered by i-CAT®) was used to evaluate the lesion three-dimensionally, operating with field of view (FOV) of 16 × 6.0 and voxel size of 0.2 mm. The resulting images enabled us to identify a homogeneous hypodensity of the lesion internally as well as its expansive aspect lingually, buccally, and inferiorly. An advanced thinning of bone cortices and complete basal displacement of the right mandibular canal were also observed. No region suggesting bone in continuity at the borderline areas of the lesion was observed either, which characterised its expansive nature (Figure 3).

The CBCT images were consistent with the diagnostic hypotheses suggested by the panoramic radiograph, which enabled us to conduct a more precise investigation of the lesion in terms of damage to the surrounding osseous structures. However, the clinical, radiographic, and tomographic characteristics were not enough to clarify the real nature of the lesion, thus making the decision-making process difficult for an initial surgical approach.

**Figure 4:**
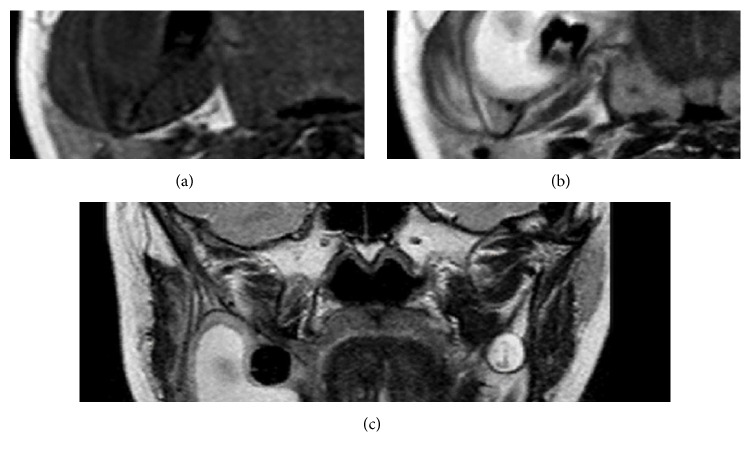
MR images showing T1 axial view (a), T2 axial view (b), and T2 coronal view (c).

In order to conduct the differential diagnostic process and evaluate the internal content of the lesion, MRI was also performed by using a Sigma Tesla machine (General Electric, Milwaukee, USA) with head coil at axial, sagittal, and coronal anatomical planes for T1- and T2-weighted images, which were obtained by using an 8-channel phased array head coil with T1 sequence (TR = 478 ms, isotropic voxel size of 0.72 mm, TE = 16 ms, FOV of 1.0 × 21.0 cm, and slice gap = 2.0 mm) and T2 sequence (TR = 6.5 ms, isotropic voxel size of 0.72 mm, TE = 90.0 ms, FOV 21 × 21 cm, and slice gap = 2.0 mm). These images have evidenced the previously viewed expansive characteristics. The T1-weighted images showed the internal intermediate signal of the lesion, which are homogeneous characteristics demonstrating the absence of calcification process and fat content. On the other hand, the T2-weighted images showed hypersignal content delimited by areas of signal ranging from intermediate to hyposignal intensity, indicating presence of liquid content (i.e., cystic content) with consequent differentiation from a solid tumour (Figure 4).

**Figure 5:**
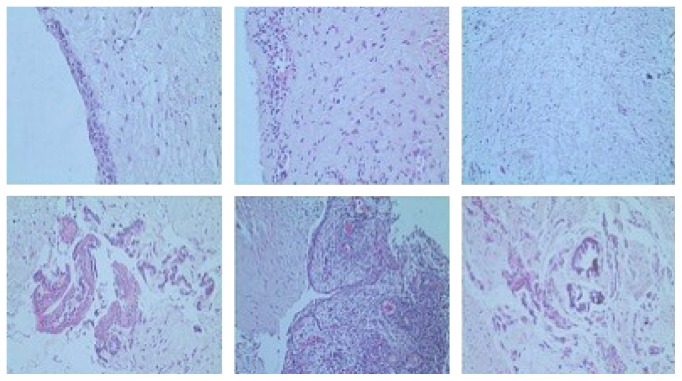
Microphotographs of the lesion stained with haematoxylin and eosin (H&E); scale bar = 80 *μ*m.

After evaluation of the MR images and aspiration punction characterising the cystic content of the lesion, an incisional biopsy was performed and a decompression device was installed. The biopsy material was sent for histopathological exam. We have observed on the laminas fragments of cystic capsule partially lined by non-keratinised stratified epithelium. In the focal area, one could note an eosinophilic staining in the epithelium as well as columnar cells in the periphery and cuboidal cells in the basal region. The capsule consisted of dense conjunctive tissue and exhibited multiple cordon-nest-like structures typical of odontogenic epithelium. In some areas, there was a tissue with myxoid appearance and in others it was possible to identify inflammatory infiltrate which was predominantly lymphoplasmocitary, ranging from discrete to moderate. Adipose tissue and hemorrhagic areas completed the histological picture, characterising the final diagnosis of dentigerous cyst as hypothesised after MRI examination (Figure 5).

**Figure 6:**
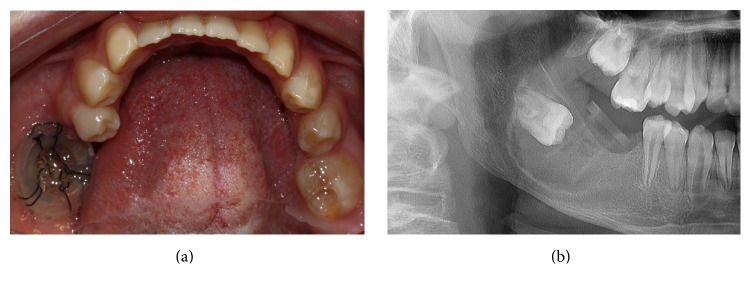
Clinical (a) and radiographic (b) aspects of the compression device following 4 months of installation.

After four months of the installation of decompression device, a new panoramic radiography was performed to evaluate the periphery of the lesion, where an area of bone sclerosis (compatible with bone neoformation) was observed, thus evidencing a tissue-repair process. With regard to the radiographic evolution of the case by comparing the images, it was observed that there was a significant decrease in the cystic volume and discrete reorientation of the eruption axes of the displaced teeth (Figure 6).

## 3. Discussion

The radiographic and tomographic aspects, including the clinical characteristics, were found to be relevant as they allowed us to determine the differential diagnosis of the present case, namely, dentigerous cyst, unicystic ameloblastoma, or KOT. This happened because these lesions are clinically and radiographically similar [3, 5–7, 9, 10].

In general, these lesions are considered diagnostic hypotheses when one observes a single lesion located in the posterior region of the mandible close to the third molar, with enhanced density at the centre, and well-delimited [5–7, 9, 10].

For this reason, MRI has played a key role in the diagnostic process by providing new information which enabled us to determine a consistent presumptive diagnosis and consequently a more coherent surgical approach [5, 7, 11].

The T1-weighted image of the lesion showed an intermediate signal, thus not being useful for determination of the content of the lesion. Nevertheless, T2-weighted image enabled us to observe an intense brightness inside the lesion, which contributed significantly to the interpretation of a probable cystic lesion rather than tumoural. These MRI data were important for us to consider the dentigerous cyst as the most probable lesion among the options raised in the differential diagnosis [7, 11].

Such information had notable repercussions on both diagnosis and initial therapeutic approach. In fact, the dentist-surgeon who performed the biopsy had considered the presumptive diagnosis of dentigerous cyst but then considered decompression as the most plausible option of treatment [12].

Regardless whether the case involves a tumoural or cystic lesion, the biopsy would be indicated anyway because the final diagnosis was based on histopathological examination as recommended in the clinical practice. The histopathological results confirmed the presumptive diagnosis of dentigerous cyst, showing that the MRI data should be considered relevant in the diagnostic process as it allows for differentiation between cystic and tumoural lesions in terms of content [13–15].

Although other lesions have a similar image pattern of dentigerous cyst on MRI, this technique helps limit substantially the broad differential diagnosis (i.e., odontogenic carcinoma in its earliest stages). Histopathology is the key element in preparing the final diagnosis [14], but MRI scans can provide a characterisation of the lesion composition and its alterations [7], thus contributing to the type of biopsy procedure to be performed. Some authors have already discussed the relevance of MRI in the dental clinic practice by highlighting its advantages and disadvantages. These authors apparently agree that the advantages overcome the disadvantages, but it is worth considering that the accessibility to MRI services becomes more difficult in the clinical routine [5, 11, 16].

Considering the real contribution of MRI to the diagnosis and treatment of the present case as well as to other cases reported in the literature, it is clear that the dentist-surgeon should consider it as an essential modern tool for optimisation of the patient treatment [5, 9, 16].

In conclusion, the benefits of MRI in the diagnostic process were found to be obvious in the present case report. The use of this complementary examination enabled the practitioner to have a safer and more efficient performance, thus optimising the treatment for the patient.
